# Abnormal Homocysteine Metabolism: An Insight of Alzheimer's Disease from DNA Methylation

**DOI:** 10.1155/2020/8438602

**Published:** 2020-09-08

**Authors:** Tingting Pi, Bo Liu, Jingshan Shi

**Affiliations:** Department of Pharmacology and the Key Laboratory of Basic Pharmacology of Ministry of Education, Zunyi Medical University, Zunyi 563000, China

## Abstract

Alzheimer's disease (AD) is a chronic neurodegenerative disease in the central nervous system that has complex pathogenesis in the elderly. The current review focuses on the epigenetic mechanisms of AD, according to the latest findings. One of the best-characterized chromatin modifications in epigenetic mechanisms is DNA methylation. Highly replicable data shows that AD occurrence is often accompanied by methylation level changes of the AD-related gene. Homocysteine (Hcy) is not only an intermediate product of one-carbon metabolism but also an important independent risk factor of AD; it can affect the cognitive function of the brain by changing the one-carbon metabolism and interfering with the DNA methylation process, resulting in cerebrovascular disease. In general, Hcy may be an environmental factor that affects AD via the DNA methylation pathway with a series of changes in AD-related substance. This review will concentrate on the relation between DNA methylation and Hcy and try to figure out their rule in the pathophysiology of AD.

## 1. Introduction

The increasing number of dementia patients in recent years is a serious problem, of which Alzheimer's disease (AD) is the most common type that accounts for an estimated 70% of dementia cases [1]. Data suggest that the prevalence of AD in people over 65 years old is approximately 10-30% and the estimated incidence is 1-3% [2]. Moreover, approximately 9.5 million people suffer from AD in China, accounting for an estimated 20% of the world in 2015 [3].

AD is a chronic neurodegenerative disease, which manifests as progressive memory loss and cognitive impairment [4, 5]. Senile plaques (SP) formed by extracellular amyloid-*β* (A*β*) peptide deposition and neurofibrillary tangles (NFTs) formed by excessive phosphorylation of intracellular tau protein constitute the hallmarks of AD [6, 7], which is also accompanied by the massive loss of neurons and synapses, as well as brain structural and functional abnormalities [8–10]. DNA methylation is an important part of epigenetics and is becoming a very attractive subject for researchers because it can shed light on unknown aspects of complex disease pathophysiology like AD. In addition, homocysteine (Hcy) is an environmental factor that seems related to AD through DNA methylation pathways.

## 2. Mechanisms

### 2.1. Alzheimer's Disease

Currently, the interaction of various factors such as genetics and environment affects the etiology and pathophysiological changes of AD [11, 12]. Multiple hypotheses are related to the pathogenesis of AD, such as the amyloid cascade hypothesis [13–15], tau protein hypothesis [16–18], cholinergic hypothesis [19], lipid metabolism disorder hypothesis [20], neuroinflammation hypothesis [21], and oxidative stress hypothesis [22], among which the amyloid cascade hypothesis and tau protein hypothesis provide the predominantly theoretical construct for AD.

**Figure 1:**
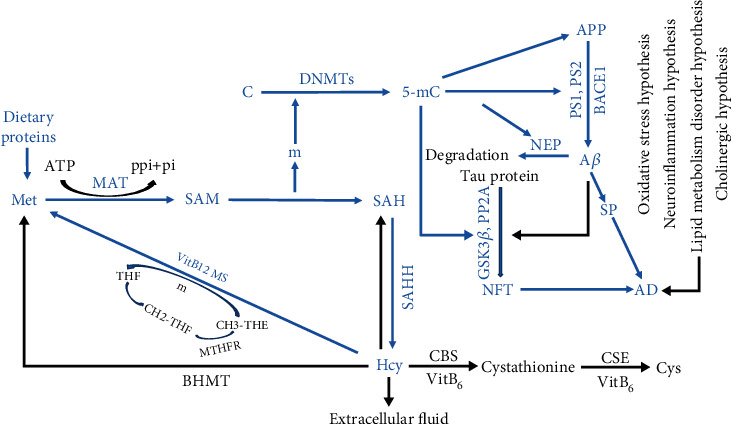
The mechanism of AD, DNA methylation and Hcy interaction. 5-mC: 5-methylcytosine; AD: Alzheimer's disease; A*β*: amyloid-*β*; APP: *β*-amyloid precursor protein; ATP: adenosine triphosphate; BACE1: *β*-secretase-1; BHMT: betaine-homocysteine methyltransferase; C: cytosine; CBS: cystathionine *β* synthase; CSE: cystathionine *γ*-lyase; Cys: cysteine; DMG: dimethylglycine; GSK3*β*: glycogen synthase kinase 3*β*; Hcy: homocysteine; m: methyl; MAT: methionine adenosyltransferase; Met: methionine; MTHFR: methylenetetrahydrofolate reductase; MS: methionine synthase; NFT: neurofibrillary tangle; NEP: neprilysin; PP2A: protein phosphatase 2A; PS1: presenilin-1; PS2: presenilin-2; SAH: S-adenosine homocysteine; SAHH: S-adenosine homocysteine hydrolase; SAM: S-adenosine methionine; SP: senile plaques; THF: tetrahydrofolate.

The amyloid cascade hypothesis indicates that the *β*-amyloid precursor protein (APP) generates A*β* peptide under the cleavage of *β*-secretase and *γ*-secretase, which eventually forms SP [23]. Previous studies have found that excessive A*β* accumulated will cause synaptic damage, glial cell overactivation [24], and inflammatory reaction [25, 26], followed by SP formation (Figure 1) [27]. A series of landmark studies states that the role of targeting A*β* peptide in the treatment of AD will delay the progression of the disease [28, 29] by inhibiting monomer aggregation and preventing the formation of toxic species [30]. Zhai et al.'s [31] study revealed that the structural origin of *β*-sheet and plaque deposition, therefore, blocking and inhibiting the transmission of A*β* from the source, may effectively inhibit A*β* deposition, which may be an important method to prevent the formation of AD.

Phosphate-containing tau protein is part of the essential components of the cytoskeleton. In normal brain cells, a single tau molecule with 2 to 3 phosphate groups is related to the stability of microtubules and the axon transport of nerve cells [32]. Abnormal hyperphosphorylation of tau protein and intraneural aggregation are hallmark features of the early development of AD-related neurofibrillary pathology. The binding capacity of hyperphosphorylated tau protein and tubulin is reduced, paired helical filaments (PHFs) aggregated; while its conversion and clearance ability are reduced, formed NFTs are the early markers of AD [33–35]. The degree of tau protein phosphorylation is the consequence of the interaction of various protein kinases (PK) and protein phosphatases (PP) in the brain (Figure 1) [36–38]. The dysregulation of PP and PK activities reduces the binding capacity and stability of tau protein to microtubules, which result in neuronal dysfunction and neurodegeneration, and further to functional defects [39–41]. A notable exception study is that tau-specific site phosphorylation protects the brain in the early stages of AD by inhibiting A*β* toxicity [42]. The hyperphosphorylation of tau is considered independent of A*β*, but the final spread of tau throughout the neocortex is driven by A*β* [7]. AD is the interaction of multiple factors and mechanisms that exert multiple effects at different stages of disease progression, which is not possible to ascribe changes in one factor. It is debatable whether the existing hypothesis fully explains to the pathogenesis of AD. Therefore, it needs further exploration to identify.

### 2.2. Homocysteine

In 1933, Vincent du Vigneaud isolated a sulfur-containing nonprotein amino acid called Hcy from bladder stones. Methionine (Met) is metabolized to S-adenosine methionine (SAM) under the action of methionine adenosyltransferase (*MAT*). SAM is one of the major methyl donors that can be converted to S-adenosine homocysteine (SAH), a methyl removed in this process, which is involved in epigenetic modifications under the action of methyltransferase [43]. SAH removes adenosine by S-adenosine homocysteine hydrolase (SAHH) to form Hcy [44], substances well associated with the methionine cycle and energy metabolism (Figure 1) [45, 46]. A clinical trial suggests that oral Met load increased plasma Hcy from 12.8 ± 1.8 to 33.3 ± 3.4 micromol·L (-1) at 4 h [47]. Consequently, excessive Met can elevate the level of Hcy, which finally results in hyperhomocysteinemia (HHcy) [48, 49].

Subsequently predominant metabolism of Hcy occurs via three pathways (Figure 1): (1) Met cycle—under the action of methionine synthase, tetrahydrofolate metabolism provides a methyl group, and Hcy is remethylated to Met with assistance of vitamin B12 (VitB12) [50, 51]; (2) transsulfuration pathway—with vitamin B6 (VitB6) as a coenzyme, Hcy and serine are condensed into cystathionine under the catalysis of cystathionine *β* synthase (CBS), followed by cystathionine catalyzed by *γ*-cystathionine lyase to produce cysteine, which is oxidized to sulfate after a series of enzyme catalysis and excreted through the urine in the form of inorganic salts [52]; and (3) direct release into the extracellular fluid—excessive Hcy is thought to be released from the intracellular fluid to the extracellular fluid through the difference in internal and external concentrations and then exported to the systemic circulation to prevent its intracellular accumulation [53–55]. VitB6, VitB12, and folic acid are the main metabolic pathways of Hcy in the methylation cycle and transsulfuration pathway, the lack of which leads to the production of HHcy. Hence, the production and metabolism balance of Hcy is essential for maintaining the body's homeostasis. Genetic factors, nutritional factors, estrogen levels, and age all affect the Hcy plasma level [56–58]. Several recent fundamental discoveries highlight important pathological roles of HHcy in many diseases [59–62]. Studies suggest that HHcy induced hypertension by promoting TLR-4-driven chronic vascular inflammation and mitochondria-mediated cell death [63]. Moreover, HHcy aggravates atherosclerosis with elevated oxidative stress and reduced S-nitrosylation level of redox-sensitive protein residues in the vasculature [64], which also as a metabolic disorder parameter is independently associated with the severity of coronary heart disease [65]. Elevated plasma total Hcy level is associated with an increased risk of neurodegenerative disease [66].

### 2.3. DNA Methylation

Epigenetics is the study of genetic changes in gene expression that is not caused by the DNA sequence changes [67]. Among them, DNA methylation is one of the best-characterized epigenetic modification, which exerts an important role in maintaining cell function, genetic imprinting, and gene expression [68, 69]. DNA methylation occurs in cytosine-phosphate-guanine (CPG) fundamental sequence with catalyzing of DNA methyltransferase enzymes (DNMTs). Specific bases in the DNA sequence and cofactor proteins are jointly involved in maintaining and regulating the methylation pattern [70–72]. DNA methylation needs a series of DNMTs [73], such as maintenance methyltransferase DNMT1 and de novo methyltransferases DNMT3a and DNMT3b [74–77]. DNMT1 maintains the continuous methylation status of DNA, which is responsible for repeated methylation during cell division [78], and DNMT3a and DNMT3b methylate DNA strands that have not been methylated, which is responsible for de novo synthesis of DNA methylation [79].

In the genome, methylated CpG sites account for approximately 70% of human genes [80]. CpG sites are located in the first exon region, gene promoter region, or intron region and regulate the expression of downstream genes [81], where the covalent bonding of the methyl group with the 5th carbon atom of cytosine is considered to be the most stable epigenetic marker [82].

## 3. DNA Methylation in Alzheimer's Disease

Epigenetics studies have found an association between DNA methylation and AD [83], which is involved in the progression of the neurodegenerative disease [84]. The earliest accumulation of A*β* reduces the overall level of 5-hydroxymethylcytosine in vitro [85], resulting in DNA hypomethylation, and affects the pathological progress of AD [86]. Furthermore, DNA methylation is associated with A*β* and NFTs [87]. PS1 is a component of the *γ*-secretase that will cleave APP to produce various A*β* [88]. Increased expression of APP and induction of hypomethylation of APP and PS1 gene promoters will increase the production of A*β* in BV-2 cells [89]. Moreover, *β*-secretase-1 (BACE1) is also hypomethylated, which affects A*β* accumulation and accelerates AD pathology [87], as well as significantly reduces DNMT1 expression in cell experiments [90]. In short, the methylation or demethylation of key enzymes will increase A*β* synthesis and reduce A*β* degradation, eventually resulting in the development of AD.

Tau phosphorylation and dephosphorylation reactions are catalyzed by glycogen synthase kinase 3*β* (GSK3*β*) and protein phosphatase 2A (PP2A), respectively; GSK3*β* and PP2A are two major kinds of enzymes that regulate hyperphosphorylated Tau. Sonawane and Chinnathambi's [91] study indicated the upregulation of GSK3*β* promoter demethylation expression and the downregulation of PP2A promoter methylation in the AD brain, both of which accelerated tau phosphorylation (Figure 1). In addition, the reduced expression of netrin-1-promoter hypermethylation may be related to memory loss [92].

DNA methylation is closely related to AD [93, 94]. Changes in DNA methylation are related to neural differentiation of the hippocampus [95], as well as across multiple brain regions. So far, DNA methylation exerts a central role in amyloid production, fibrogenesis, inflammation, and oxidative pathways. All the above studies suggest that DNA methylation is involved in the AD-related molecular mechanism [96].

## 4. Homocysteine in Alzheimer's Disease

With increasing age, the risk of AD increases under the interaction of genetic and environmental factors (obesity, smoking, and an unhealthy lifestyle) [97], of which Hcy is a risk factor of AD. Several studies are indicating that high Hcy concentrations cause cognitive dysfunction [98] and might be associated with dementia [99–101].

HHcy may promote dementia through a variety of mechanisms, including cerebral microangiopathy, endothelial dysfunction, oxidative stress, neuronal damage, and A*β*-mediated enhancement of vascular toxicity, neurotoxicity, and apoptosis [102]. The brain of AD patients is accompanied by cerebrovascular disease [103], and studies show a long-term high Hcy diet severely induces microbleeds, which may be the cause of memory deficits [104]. Although elevated Hcy does not induce lipid peroxidation in the whole brain of rats, similar physiological changes in levels are observed in both malondialdehyde (MDA) and superoxide anion (SOA), resulting in oxidative stress [105]. Not only does Hcy can increase the activity of MMP-9 and MMP-2 but also reduce the activity of arginase. Meanwhile, it is accompanied by nitrosative stress reaction that destroys the integrity of the blood-brain barrier (BBB), leading to cerebrovascular permeability and neurodegeneration [106, 107]. Lin et al.'s [108] study suggests that Hcy can affect nerve cell proliferation and A*β* deposit formation by inducing an increase in intracellular SAH [109–111]. Additionally, DNA damage-related genes are significantly upregulated and trigger oxidative and genotoxic stress [112]. Since high Hcy levels are a metabolic risk factor for neurodegenerative diseases, diet-induced Hcy levels not only increase aggravate Tau neuropathology in H-TAU mice but also affect synaptic integrity, neuroinflammation, and cognition function [113]. Moreover, the AD transgenic mouse model shows that A*β* content in cerebral blood vessels increased significantly, neurons died, and DNA damage of hippocampal neurons further reduce cognitive ability [114]. Excessive deposition of hyperphosphorylated Tau and neuropathy caused by synaptic inactivation lesions are also associated with the elevated Hcy level [115–117].

Hcy level changes AD development by inducing neuronal DNA damage, neuroinflammation, apoptosis, and autophagy abnormalities [117–119]. Genetic variation affects the relevant genes, which advances the age of onset and accelerates cognitive function decline [120, 121].

## 5. DNA Methylation and Homocysteine in Alzheimer's Disease

Dementia-like symptoms caused by HHcy are related to abnormal methylation and gene expression disorders [122]. One study found that HHcy can reduce the methylation level and increase cell damage by inhibiting the protein expression and enzyme activity of DNMT1, DNMT3A, and DNMT3B in the hippocampal neural stem cells of raw rat [123]. Another study also illustrates that HHcy enhances DNA damage by inducing methyl donor deficiency and disrupting DNA repair, resulting in neuronal cell death [124]. In addition, the upregulation of the 5lo enzyme pathway leads to hypomethylation of 5loDNA and promotes the formation of A*β* [125]. HHcy can decrease the activity of methylenetetrahydrofolate reductase (MTHFR) and tight connexin expression, while SAHH expression, BBB permeability, and oxidative stress are increased with DNA methyltransferase upregulation, resulting in neurodegeneration and synaptic toxicity [126]. Most importantly, the Met cycle and transsulfuration pathway are related to VitB family folic acid [127, 128].

Folic acid is involved in the regulation of one-carbon metabolism and methylation. In addition to this, the active form of folic acid is 5-methyltetrahydrofolate, which is a methyl donor for the remethylation of Hcy. HHcy is elicited by low folic acid, which damages hippocampal neurons and is an important factor of the high incidence of dementia in the elderly [129]. Furthermore, folic acid is not only positively related to the DNA methylation level of the cognitive impairment elderly but also related to the intensity of DNA methylation [130]. MTHFR is involved in folate metabolism, and the high level of Hcy caused by MTHFR deficiency will reduce the expression and methylation level of PP2A and leucine carboxylmethyltransferase 1 (LCMT1), resulting in tau dephosphorylation [131].

HHcy is a risk factor for AD and is also associated with VitB12 deficiency [132]. The accumulation of Hcy induced by VitB deficiency may impair the “methylation potential,” resulting in the upregulation of PS1, BACE, and increased A*β* [133, 134]. Several studies have implicated that the plasma Hcy level in the AD group increased while the folate and VitB12 levels decreased [135–138]. Moreover, abnormal Hcy metabolism causes plasma folic acid and VitB12 deficiency [139–142], which in turn affects the methylation level of AD-related genes via participating in AD development [143]. Mice lacking folic acid and VitB diets will have increased Hcy levels, A*β* levels, and tau phosphorylation, which is also accompanied by hypomethylation of the Alox5 promoter [144]. High Hcy-induced SAH increases [123, 124], and the SAM/SAH ratio decreases, both of which are related to the inhibition of methyltransferase [145]. Methylation analysis also further demonstrates the correlation between the SAM/Hcy cycle and DNA methylation, involved in PS1 and BACE1 methylation [141]. SAM is the predominant methyl donor; Scarpa et al. [145] analyzed the effect of SAM administration on the expression of 588 central nervous system genes in nerve cells and showed that among the seven genes treated by SAM, three genes had DNA methylation upregulated and four genes had DNA methylation downregulated [146]. SAM can regulate its products to take part in the methylation status of APP genes, which affects the formation of A*β* [147] by increasing APP and PS1 proteins expression; it can also induce hypomethylation of APP and PS1 gene promoters and increase A*β* production in BV-2 cells [148]. In short, Hcy can change the DNA methylation levels of key metabolic enzymes and cause brain damage [149].

## 6. Future Directions

Possible mechanisms for Hcy to induce AD are shown in Figure 1; high levels of Met intake produce excessive Hcy in the body which metabolizes through the Met cycle, during which the generated methyl adds to the five-bit carbon atom of the cytosine under the action of DNMTs, causing the methylation levels of the AD-related genes to change. The changes in methylation levels in turn affect the expression of the gene, resulting in the occurrence of AD. At the same time, excess accumulation of Hcy is regeneration to methionine under the action of methionine synthesis enzyme and VitB12, finally producing the Hcy. As a result, high levels of Hcy may induce AD along with changes in methylation levels of AD-related genes.

Unbalanced nutritional intake will not only increase Hcy levels but also affect DNA methylation and gene expression. At present, most researchers focus on the effect of Hcy on AD symptoms, rather than on molecular mechanisms. At the molecular level, studying the regulatory mechanism of Hcy and its metabolites on the expression of related genes in AD patients helps determine the appropriate nutritional requirements. Preventing the increase of the Hcy level caused by the imbalance of nutrition intake can either avoid or arrest the occurrence and aggravation of AD.

As AD progresses, treatment becomes difficult with little effect [150, 151]; the research and development of drugs also consume a lot of manpower and material resources [152]. So far, the main drugs used in AD treatment are donepezil, rivastigmine, galantamine, and memantine, which can only relieve symptoms but can not cure and reverse the development of AD [153–156]. In addition, some drugs must be used in combination to achieve the best therapeutic effect, which is also accompanied by increasing the risk of various adverse reactions [157]. Since 2003, the FDA has not approved a new drug for the treatment of AD [158]. Therefore, early diagnosis and treatment are essential. The current clinical early diagnosis depends on clinical observation, and cognitive testing is the first step to diagnose the complex disease characteristics in AD, which is time-consuming and has limitations. More definite diagnosis requires imaging (MRI or PET scan) or invasive lumbar puncture to measure CSF markers which is expensive. Thus, efficient diagnostic methods and early disease biomarkers are essential for the prevention and treatment of early AD [159].

Several researchers have reported that plasma Hcy levels are usually elevated in patients with AD [160]. HHcy is closely related to cortical atrophy and more severe cognitive decline [161, 162]. The high plasma Hcy concentrations are significantly associated with mild cognitive impairment (MCI) and AD, which is more strongly correlated with AD patients as compared to patients with MCI [163]. A meta-analysis included 34 studies with 9397 subjects and demonstrated a causal link between plasma total Hcy and the risk factor of AD [164]. More than 40% of patients with AD are associated with a high Hcy level in the plasma, which is associated with a more rapid neural atrophy than those with normal levels of Hcy [165]. Moreover, HHcy levels can predict a cognitive decline in healthy elderly patients [166, 167]. Therefore, HHcy also has the potential to predict AD, and preventing Hcy-induced neurotoxicity may become a novel strategy for AD prevention and treatment.

DNA methylation alteration in the hippocampus of AD patients occurs in specific regulatory regions that are critical to neurodifferentiation; this supports the idea that hippocampus neurogenesis may play a role in AD through epigenetic mechanisms [168]. The current findings suggest that the epigenetic modulation of DNA is vulnerable to the state of neurodegenerative diseases [169]. Moreover, brain DNA methylation is associated with AD pathology in multiple AD loci, and the results further prove that the destruction of DNA methylation is involved in the pathological process of AD [170]. Many researches have shown that DNA methylation is a useful marker for screening individuals at the risk of AD [171]. Therefore, AD-related gene methylation levels are a convenient and useful biomarker for AD diagnosing [172–174].

Proper nutrition not only changes Hcy levels but also prevents the development of AD and reduces cognitive impairment. Hcy levels may develop into AD biomarkers for diagnosis; moreover, factors that affect Hcy's production and metabolism not only increase Hcy levels but also affect DNA methylation levels of AD-related genes. Studying the mechanisms of DNA methylation in AD can help to explore the etiology and pathogenesis of AD, which can also be a very useful tool for researchers to identify AD biomarkers and even play an important role in early screening of patients in the future. Meanwhile, effective measures to reduce Hcy levels and DNA methylation will provide new ideas for the prevention and treatment of AD.

## Data Availability

No data were used to support this study.
